# A Systematic Investigation of the Kinetic Models Applied to the Transport Behaviors of Aromatic Solvents in Unfilled Hydrogenated Nitrile Rubber/Ethylene Propylene Diene Monomer Composites

**DOI:** 10.3390/polym16070892

**Published:** 2024-03-25

**Authors:** Susu Liu, Yiran Jing, Guangyong Liu

**Affiliations:** Key Laboratory of Rubber-Plastics, Ministry of Education, Qingdao University of Science & Technology, Qingdao 266042, China; lss8130@163.com (S.L.); 17863957197@163.com (Y.J.)

**Keywords:** Korsmeyer–Peppas model, Peppas–Sahlin model, rubber–solvent system, transport time

## Abstract

Kinetic models of solvent transport behaviors are widely used in rubber–solvent systems, and some key points are still worthy of attention. In this work, the Korsmeyer–Peppas and Peppas–Sahlin models were chosen to fit the transport behaviors of three aromatic solvents, benzene, toluene and p-xylene, in the hydrogenated nitrile rubber (HNBR)/ethylene propylene diene monomer (EPDM)-based vulcanizates. The different effects of the various selected transport times (t_i_) used for fitting on the results of the mathematical models were compared. Moreover, a method to obtain the n parameter for the Korsmeyer–Peppas model and the m parameter for the Peppas–Sahlin model at t_i_ = 0 was discussed. It was found that the differences in values of t_i_ greatly influenced the impact on the fitting results of all the parameters for the two models. In addition, the n parameter for the Korsmeyer–Peppas model along with the m parameter for the Peppas–Sahlin model, which can characterize the transport mechanism, showed differing applicability. But the n and m parameters at t_i_ = 0 obtained by linear fitting showed similar rules with some differences in values. These discussions give important guidance for the application of kinetic transport models in rubber–solvent systems.

## 1. Introduction

Rubber is an important polymer material with high elasticity at room temperature and one of the crucial materials used by human beings. It is widely used in the automobile, petroleum, machinery industries, and so on [1]. The solvent resistance of rubber plays an important role in the application of rubber products that are often in contact with liquid environments [2,3]. The entry of small molecular liquids greatly reduces the performance of rubber products and thus shortens the service life, making an investigation of the transport process of solvent molecules in rubber more meaningful [4,5]. With further investigation of the solvent resistance of rubber, some kinetic transport models, such as the Korsmeyer–Peppas and Peppas–Sahlin models, which were originally used in the pharmaceutical field, have been frequently applied to rubber–solvent systems to characterize the transport mechanism [6]. The equations of the Korsmeyer–Peppas model (Equation (1)) and Peppas–Sahlin model (Equation (3)) are listed as follows [7,8],

(1) $$\frac{Q_{t}}{Q_{\infty }}={\mathrm{kK}}_{p}t^{n}$$

where Q_t_ is the mole% uptake of solvent for rubber vulcanizate at time “t”, which can be calculated from Equation (2). Q_∞_ is the value of Q_t_ when the swelling of rubber reaches an equilibrium state, k is a kinetic constant characteristic of the rubber/solvent system, kK_p_ is a release constant combining structural and geometric characteristics, and n represents the transport mode. When n = 0.5, the transport mechanism is considered to be the Fick diffusion mode, which refers to a situation where the relaxation rate of the polymer chain is higher than the diffusion of the solvent molecules. The transport mode is classified as non-Fickian while n = 1, with the penetration rate of the solvent molecules higher than the chain relaxation rate. In addition, while the n value remains in the range from 0.5 to 1.0, the mode is matched with the anomalous mode [9,10].

(2) $$Q_{t}=\frac{M_{S}/M_{\mathrm{mol}}}{M_{R}}\times 100$$

where M_S_ is the mass of solvent entering the rubber, M_mol_ is the molar mass of the solvent, and M_R_ is the initial mass of the rubber.

The Peppas–Sahlin model is expressed as follows,

(3) $$\frac{Q_{t}}{Q_{\infty }}=K_{f}t^{m}+K_{r}t^{2m}$$

where K_f_ is the Fickian diffusion contribution coefficient, K_r_ is the relaxation contribution coefficient, and m is purely the Fickian diffusion exponent, which has the same boundary as the n parameter for the film shape [11].

With the in-depth study of the solvent resistance of rubber, these two mathematical models are widely used to explain the transport mechanism of solvents in rubber. Unnikrishnan studied the transport behavior of aromatic solvents in natural rubber and chose the Korsmeyer–Peppas model to characterize the transport mechanism, and it was found that values of n were mostly between 0.5 and 1.0, indicating the anomalous mode [12]. According to Aminabhavi, the n values of the Korsmeyer–Peppas model for an n-alkane-thermoplastic rubber system were also higher than 0.5 and lower than 1.0, but closer to 0.5 [13]. And a similar phenomenon in the aromatic solvent linear low-density polyethylene/ethylene vinyl acetate blend system was found by Moly [14]. Both Korsmeyer–Peppas and Peppas–Sahlin models were used to research the diffusion mechanism of organic solvents in a natural rubber/nitrile rubber blend by Maria, and the n values indicated the anomalous mode. In addition, the values of K_f_ were always higher than K_r_, suggesting that release was mainly determined by solvent diffusion [6]. It should be noted that the phenomenon K_f_ >> K_r_ occurred in most research work about rubber–solvent systems [15,16,17]. In particular, in the study of cyclohexane in nanosilica-reinforced ethylene propylene diene monomer/isobutylene isoprene rubber, Neelesh Ashok found that the K_f_ values were much lower than the K_r_ values, and the n values were always higher than 1.0 [18]. However, all the above work for rubber–solvent systems did not elaborate on or limit the transport time or degree of solvent used for mathematical fitting, which were different from the 60% degree of drug release [19]. Only a combination of accurate and precise data with models accurately depicting the physical situation will provide an insight into the actual transport mechanism. Therefore, the determination of the transport time or the degree of solvent diffusing into the rubber used for model fitting is vital in order to obtain the correct mechanism.

**Figure 1:**
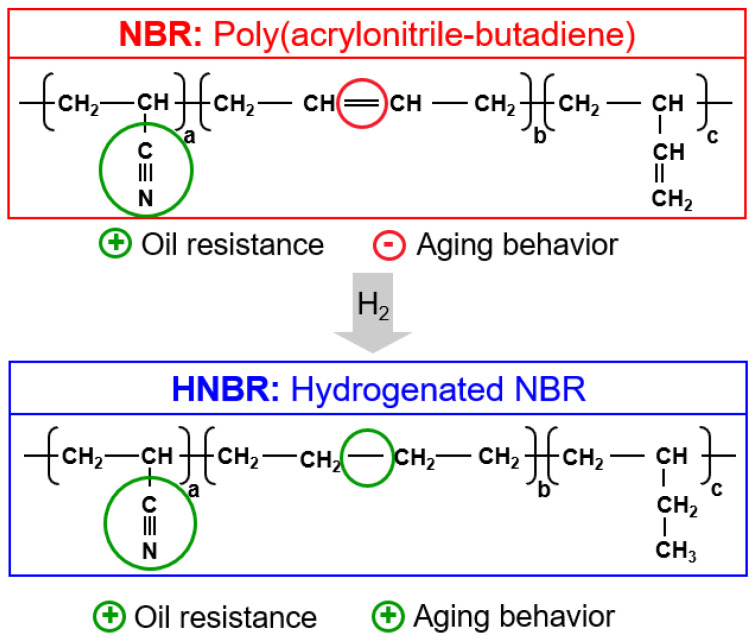
Hydrogenation of NBR to HNBR with associated improvement of ageing performance.

Hydrogenated nitrile rubber (HNBR) is a kind of special rubber prepared by selective hydrogenation on the nitrile rubber (NBR) macromolecular chains (Figure 1), which has good thermal and oxygen aging resistance, ozone resistance, and chemical medium resistance [20,21]. HNBR is a copolymer based on acrylonitrile and 1,3-butadiene, and for special grades additional termonomers are incorporated into the polymer backbone. HNBR is produced by homogeneous or heterogeneous catalytic hydrogenation of NBR, typically in a solution process. It also shows excellent dynamic and mechanical properties because of strain-induced crystallization upon orientation of poly(methylene) sequences in the polymer backbone. Hence, HNBR can be widely used as seals, hoses, belts and gaskets in automotive and industrial applications.

**Figure 2:**
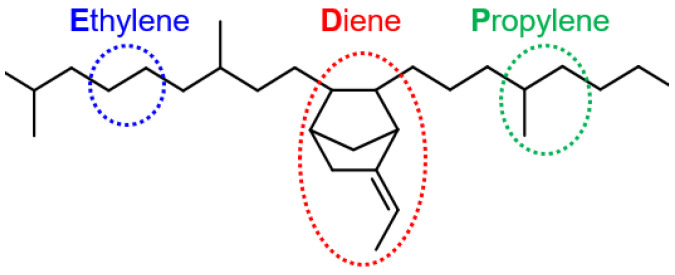
Macromolecular structure of EPDM.

Ethylene propylene diene monomer (EPDM) is copolymerized by ethylene, propylene and the non-conjugated diene (Figure 2), which shows good heat resistance and weather resistance [22,23]. It is produced via insertion polymerization employing Ziegler–Natta catalysis, typically an in-solution process. EPDM has a fully saturated hydrocarbon main chain with a low level of unsaturation in the side groups.

As a result, EPDM has a very good resistance against ozone, oxygen and irradiation, and against aqueous systems and a broad range of acidic and alkaline chemicals. Therefore, the combination of HNBR and EPDM should ensure that the blend has excellent integrative properties [24].

The transport behaviors of molecules in HNBR/EPDM composites caused by oil swell have rarely been studied, yet it plays a decisive role in the applications involving contact with chemical media. Moreover, the volume expansion of HNBR/EPDM composites resulting from chemicals leads to a rapid decline in properties [25,26]. The study of the transport behaviors of small molecules in rubber composites characterized by Korsmeyer–Peppas and Peppas–Sahlin models makes a great contribution to the practical application. In addition, the transport behaviors of various organic solvents through polymers is of great importance and it plays a vital role in the selection of rubber composites for specific applications [27]. Diffusion is a kinetic process that depends on the nature of the rubber or rubber blends, crosslink density, penetrant size and others. Stephen studied the transport behaviors of rubber blends based on NR and halogenated nitrile butadiene using aromatic solvents (benzene, toluene, and xylene) and reported a decrease in the diffusion coefficient as a function of an increase in the weight percentage of filler [28]. James studied the transport properties of SBR- and PMMA-based IPN and found that an increase in crosslinker level lowers the solvent uptake. The experimental results of Qt versus the square root of time were analyzed using the Peppas–Sahlin and Korsmeyer–Peppas models, which fitted the Peppas–Sahlin model well [17]. Lovely analyzed the swelling behavior of isora/natural rubber composites and observed that the uptake of aromatic solvents was higher than aliphatic solvents for the composites [29]. Thomas studied the diffusion and transport of organic solvents through lignin-filled NR composites and reported that the mechanism of transport followed the Fickian law of diffusion and the crosslinked network structure could not be broken down completely by any solvent [30].

However, limited research work is available on the transport behaviors of solvents in rubber blends, especially for HNBR/EPDM composites, even though it is very important in the area of solvent-resistant rubbers. In our recent work, the transport behaviors of ester solvents were studied through the calculation of sorption and permeation coefficients. The sorption and permeation coefficients showed two-stage upward linear relationships with the HNBR concentration, which could be attributed to the sea phase in the blend playing a decisive role in the transport behaviors of the solvents. Moreover, the trend of the selective adsorption of the solvent was more obvious in the permeating process than at the final equilibrium swelling state. The Korsmeyer–Peppas model and Peppas–Sahlin model were selected to explain their transport behaviors, indicating that the release of the ester solvents is mainly due to solvent diffusion. Moreover, the values of fitting degree (R^2^) for the Peppas–Sahlin model were always higher than those of the Korsmeyer–Peppas model [31]. These studies, however, only provide a preliminary prediction guide for the swelling behavior for such HNBR/EPDM composites.

In this work, the effect of selected transport time or degree on the fitting results of the Korsmeyer–Peppas and Peppas–Sahlin models was studied in depth. The transport processes of three aromatic solvents, benzene, toluene and p-xylene, in the HNBR/EPDM blends with eleven different blending ratios provide a large amount of experimental data support. The n parameter for the Korsmeyer–Peppas model and the m parameter for the Peppas–Sahlin model, which can characterize the transport mode of solvent molecules, were compared in depth, and some interesting rules were found, which can provide good guidance for the application of Korsmeyer–Peppas and Peppas–Sahlin models in rubber–solvent systems.

## 2. Experiment

### 2.1. Materials

The HNBR (acrylonitrile content: 34 wt%) and EPDM (ethylene content: 48 wt%; ENB content: 4.1 wt%) used in this work were supplied by ARLANXEO (The Hague, The Netherlands). The curing systems consisting of dicumyl peroxide (DCP) and triallyl isocyanurate (TAIC) were supplied by Arkema (Paris, France). The three aromatic solvents were purchased from Shanghai Macklin Biochemical Co., Ltd. (Shanghai, China). The basic physical properties of these solvents are shown in Table 1.

### 2.2. Preparation of Samples

In order to reduce the influence of other factors, only the vulcanization system was under consideration, and the formulations as listed in Table 2 were as follows. HNBR and EPDM were first mixed in an internal mixer (Shanghai KCCK Co., Ltd., XSM-500, Shanghai, China) at a rotor speed of 80 rpm for 3 min at 60 °C. Then, uniformly dispersed HNBR/EPDM blends were obtained by adding vulcanizing system through a two-roll mill (BOLON Precision Testing Machines Co., Ltd., BL-6175, Dongguan, China) at 50 °C with a roller speed ratio of 1:1.2. Finally, the sample curing was carried out on a hydraulic press at 175 °C and t_90_ + 5 min as curing time determined by the moving die rheometer to make rubber sheets of 2 mm thickness.

### 2.3. Swelling Experiment

The transport behaviors of the aromatic solvents depend not only on the rubber–solvent interaction but also on the network density in accordance with the Flory–Rehner theory. Furthermore, the shape or thickness of the rubber sample can affect the transport time and path of the solvent to a certain extent. Therefore, the vulcanized sheets were cut into circular samples (1.0–1.2 g for each sample) with the same shape to ensure they had the same area effect. These samples (3–5 per test) were first weighed for the initial weight, then immersed in the three aromatic solvents for the diffusion experiments. At specific intervals, the test samples were taken out of the liquid containers, and extra solvent on the surface was wiped out quickly with filter paper. Then, the samples were weighed immediately. After weighing, the samples were placed back into the original test bottles. Such operations were conducted several times until reaching equilibrium swelling and then weighed again to obtain the final weight.

Importantly, any low molecular weight substances and uncrosslinked components should be extracted from the rubber samples. Such extraction treatment before swelling tests was carried out to avoid errors from fluid extraction effects. And moreover, the volume of solvent available to the rubber sample for the swelling should be large enough in order to avoid the effect of solvent shortage.

## 3. Results and Discussion

### 3.1. Transport Process of Aromatic Solvents in HNBR/EPDM Blends

**Figure 3:**
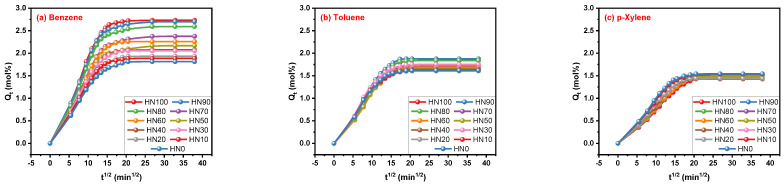
Q_t_ curves of (**a**) benzene, (**b**) toluene and (**c**) p-xylene transporting in HNBR/EPDM blends.

The transport processes of the three aromatic solvents, benzene, toluene and p-xylene in the HNBR/EPDM blends can be characterized by the Q_t_ curves shown in Figure 3. This reveals that the Q_t_ values increase gradually with the extension of time and the upward trends of Q_t_ decrease with time until swelling equilibrium is reached. The maximum concentration gradient occurs when the solvents begin to enter the rubber blends, and the blends reach swelling equilibrium state while the force of the solvent entering is equal to the retraction force of the molecular chains of the HNBR/EPDM blends produced by expansion. As can be seen in Figure 3a–c, the difference in the solvents absorbed by the HNBR/EPDM blends with different blending ratios gradually decreased from benzene to p-xylene, which may be due to the different interactions between the HNBR/EPDM blends and the three different solvents. For the purpose of making the topic more focused, the specific interactions between the solvents and the rubber blends, and as well the influence of the morphology of the HNBR/EPDM blends is not discussed here. It is not detrimental to the conclusion of this work and some prior studies were carried out. Furthermore, the selection of t^1/2^ for abscissa provides a better comparison with the same type of research.

### 3.2. Fitting Process

**Figure 4:**
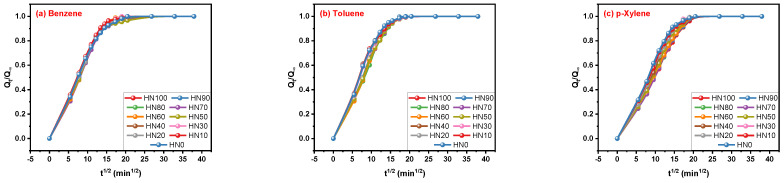
Q_t_/Q_∞_ curves of (**a**) benzene, (**b**) toluene and (**c**) p-xylene transporting in HNBR/EPDM blends.

**Figure 5:**
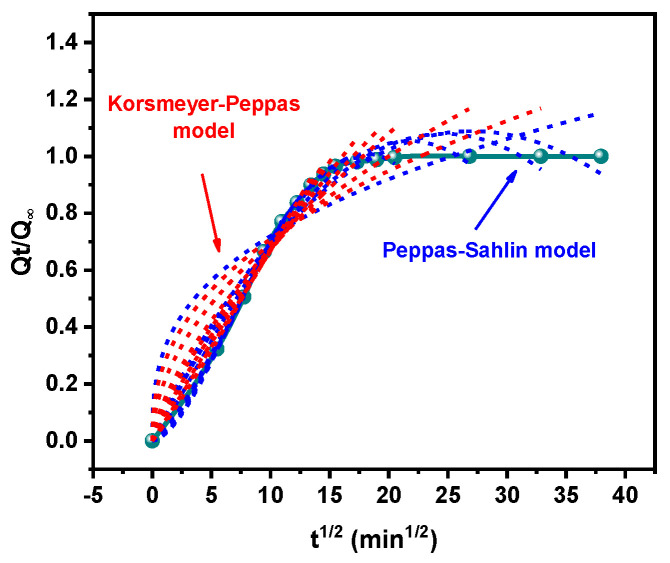
Fitting process of Korsmeyer–Peppas and Peppas–Sahlin models with different time points (taking benzene transportation in HNBR as an example).

Through the mathematical equations of the Korsmeyer–Peppas and Peppas–Sahlin models, it was found that the correlation between Q_t_/Q_∞_ and time should be obtained first. The interesting phenomenon is that the dispersion degrees of the Q_t_/Q_∞_ curves declined from Figure 4a–c, which was contrary to those of the Q_t_ curves depicted in Figure 3. On the basis of the results shown in Figure 4, the fitting of the two models was further carried out in the following Figure 5.

As shown in Figure 5, all the time points of the tested weights of the HNBR/EPDM blend sample swelling in benzene were used for fitting by the Korsmeyer–Peppas and Peppas–Sahlin models in order to study the effect of the selected transport time on the fitting results in depth. And for a more concise representation, t_i_ was defined as the selected transport time used for fitting. It is worth mentioning here that the t_i_ should be distinguished from the true transport time because it is the choice of time point that determines the fitting results of mathematical models, even if they are the same value. Based on the fitting process in Figure 5, all the experiment data was fitted by the two mathematical models, involving a great deal of calculation process, which was not fully displayed for the sake of brevity. The derived fitting results are discussed in the following sections.

### 3.3. Results of Fitting by Korsmeyer–Peppas Model

**Figure 6:**
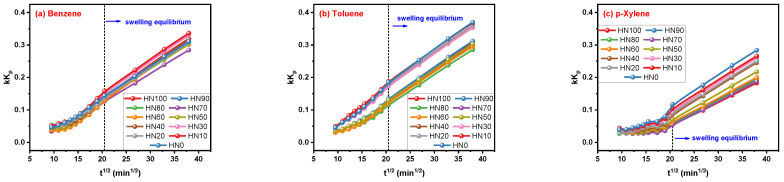
Correlation of kK_p_ parameters for Korsmeyer–Peppas model and t_i_.

The plots in Figure 6a–c clearly explain the trend that values of the kK_p_ parameter for the Korsmeyer–Peppas model always increase with the increasing t_i_ value. The tendency of kK_p_ growing with t_i_ before and after the swelling equilibrium state is different with the smooth front and steep rear segments. Although the kK_p_ parameter is not often discussed in rubber–solvent systems on account of the nonexistence of a numerical boundary, it was found that the correlation of t_i_ and the fitting results cannot be ignored. Now, let us turn our attention to the other parameter, n, of the Korsmeyer–Peppas model.

**Figure 7:**
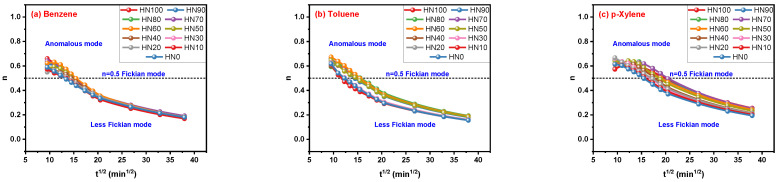
Correlation of n parameter for Korsmeyer–Peppas model with t_i_.

By mathematical fitting, the increase in t_i_ values led to the reduction in n values in Figure 7a–c. When t_i_ values are relatively low, the values of the n parameter are higher than 0.5 (above the dotted line) and lower than 1.0, indicating the transport mode of solvent molecules in HNBR/EPDM blends is the anomalous mode. The less-Fickian mode (below the dotted line) is matched when t_i_ values are relatively high. Now, an important phenomenon that has been discovered is that different t_i_ values used by the Korsmeyer–Peppas model obtain different transport modes determined by n values. The importance of t_i_ is well reflected in the achievement of the n parameter. A random selection of time points cannot obtain reasonable results, a fact to which attention should be paid.

### 3.4. Results of Fitting by Peppas–Sahlin Model

**Figure 8:**
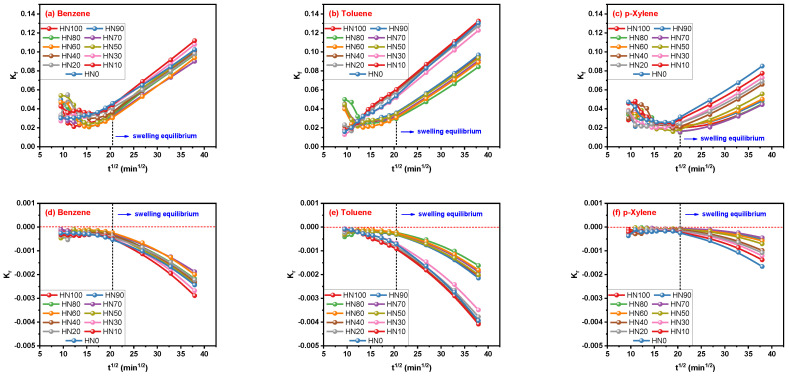
Correlation of K_f_ and K_r_ parameters for Peppas–Sahlin model with t_i_.

On careful observation of Figure 8a–c, the values of K_f_ show a declining tendency at first and then increase with the increasing t_i_ in all of the three aromatic solvent–HNBR/EPDM blend systems. Upward and then downward trends of K_r_ values were found with the increase in t_i_ values in Figure 8d–f, which was opposite to K_f_. In addition, the values of K_f_ were always higher than zero, and values of K_r_ were always lower than zero for all the solvent–blend systems, which indicates that the release of solvent molecules is mainly controlled by diffusion. Coincidently, it is difficult to find a situation where the K_r_ value is higher than the K_f_ value in the previous application of the Peppas–Sahlin model on solvent–rubber systems, which does not always match the mechanism determined by the n parameter for the Korsmeyer–Peppas model. And the law that K_f_ values are always higher than that of K_r_ is established in Figure 8, even though the values of both K_f_ and K_r_ vary with t_i_, forcing us to consider the credibility of the comparison between these two parameters, or if all ordinary rubber–solvent systems conform to this law.

**Figure 9:**
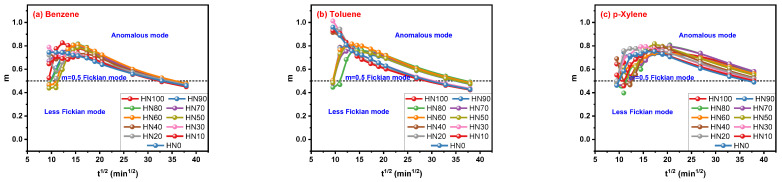
Correlation of m parameter for Peppas–Sahlin model with t_i_.

It is obvious that the m parameter values of the Peppas–Sahlin model are more consistent in the numerical data than the n parameter for the Korsmeyer–Peppas model, although m values still vary with t_i_ values. Almost all the values of m are between 0.5 and 1.0 before reaching equilibrium swelling in the HNBR/EPDM blends, indicating the anomalous mode. Then, the transport mechanism determined by the m parameter is the same as that obtained from K_f_ and K_r_. It should be noted that m will be less than 0.5 with an increase in t_i_ after the swelling equilibrium state is reached, so a too large t_i_ value is not favorable for obtaining the transport mechanism of solvent in rubber. Combined with the information in Figure 8 and Figure 9, one can draw a general conclusion that the transport mode of the three aromatic solvents in the HNBR/EPDM blends is an anomalous mode dominated by solvent diffusion. The specific fitting degree of both the Korsmeyer–Peppas and Peppas–Sahlin models has not been discussed due to too much data, but a clear phenomenon is that the latter always has the higher fitting degree.

### 3.5. A Way to Obtain n and m Parameters at t_i_ = 0

**Figure 10:**
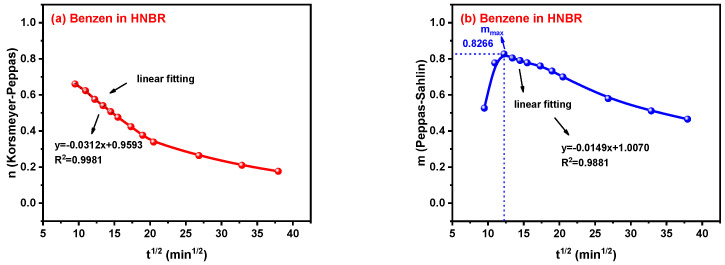
Fitting process of n parameter for Korsmeyer–Sahlin model and m parameter for Peppas–Sahlin model.

Taking the transport process of benzene in the HNBR/EPDM blends as an example, an obvious linear relationship can be found in the initial stage of the n curves by observation, and high fitting degrees were achieved after linear fitting. The n value is the intercept of the fitting line when the value of x, that is t_i_, is zero, which is named n_linear_. Moreover, the linear part of the m curve occurs in the initial descending region after the turning point of the rise and fall. The linear fitting of m curves was also carried out and the intercept was named m_linear_, which will be compared with n_linear_ in the following content. Based on this interesting correlation, a way to obtain the n and m parameters at t_i_ = 0 is assumed. And the special turning point of the m curve, named m_max_, is also selected for further discussion. The process of obtaining n_linear_, m_linear_ and m_max_ of all the systems follows the way shown in Figure 10 and the results are discussed in the following sections.

**Figure 11:**
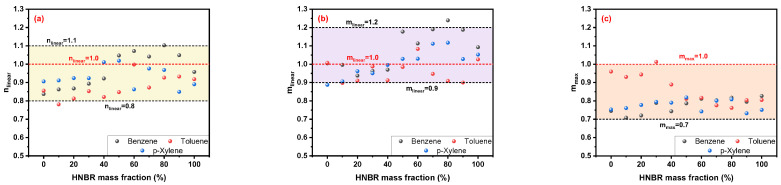
Linear fitting results of (**a**) n_linear_ for Korsmeyer–Peppas model, (**b**) m_linear_ for Peppas–Sahlin model and (**c**) the special turning point m_max_.

**Figure 12:**
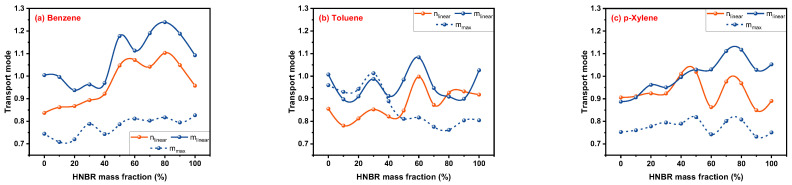
Comparison of n_linear_, m_linear_ and m_max_.

As shown in Figure 11a,b, the linear fitting results of the n parameter for the Korsmeyer–Peppas model are mostly between 0.8 and 1.1, and that of the m parameter for Peppas–Sahlin model is higher than 0.9 and lower than 1.2. In addition, the m_max_ values are generally greater than 0.7 and less than 1.0. Some values of nlinear and mlinear are close to or even higher than 1.0, representing the non-Fickian transport mode, which is inconsistent with the conclusion obtained in Figure 9. Furthermore, these three parameters for the same solvent percolating into the HNBR/EPDM blends are compared in Figure 12.

Although there is no satisfying feedback on the values of n_liner_ and m_linear_ determined by linear fitting, an exciting phenomenon was found when comparing them for the same rubber–solvent system. From Figure 12a–c, it can be seen that the shapes of the n_linear_ and m_linear_ curves are surprisingly similar, which is not the case in Figure 7 and Figure 9. This is a meaningful discovery for the comparison between the Korsmeyer–Peppas and Peppas–Sahlin models when applied to rubber–solvent systems, which find the similarity law of n and m parameters that represent the same meaning in two different mathematical models, even if some problems need to be further studied. In addition, no clear connection has been found between m_linear_ and m_max_; they are only similar in shape in Figure 12c.

## 4. Conclusions

The transport mechanism plays an important role in the study of the transport behavior of solvent molecules in HNBR/EPDM blends and the liquid resistance of the blends. All the parameters of the two kinetic transport models, Korsmeyer–Peppas and Peppas–Sahlin, vary with the selected transport time, t_i_, although their trends are different. Especially, for the n and m parameters which can characterize the transport mechanism, different t_i_ values can take on different transport mechanisms, meaning the effect of transport time cannot be ignored. Although the models are different, the values of n_linear_ and m_linear_, obtained by linear fitting in this paper, show similar rules, which provide an idea for future research in this field. On the whole, the Peppas–Sahlin model shows better usability than Korsmeyer–Peppas model in evaluating the transport of aromatic solvents in HNBR/EPDM blends.

There is no doubt that finding a suitable transport time for mathematical fitting is not only conducive for judging the mechanism of the solvent transport, but would also provide a basis for the discussion of the use of the specific model. These findings should be paid attention to and be expected to make some contribution to future studies of solvent transport behavior in rubber–solvent systems.

## Figures and Tables

**Table 1:** Basic information of benzene, toluene and p-xylene.

Solvent	Chemical Formula	Molar Mass (g/mol)	Density (g/cm^3^)	Molar Volume (cm^3^/mol)	Structure
Benzene	C_6_H_6_	78.11	0.87	89.50	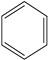
Toluene	C_7_H_8_	92.14	0.86	106.60	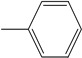
p-Xylene	C_8_H_10_	106.17	0.88	121.10	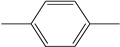

**Table 2:** Formulations of HNBR/EPDM blends.

Materials	HN100	HN90	HN80	HN70	HN60	HN50	HN40	HN30	HN20	HN10	HN0
HNBR	100	90	80	70	60	50	40	30	20	10	0
EPDM	0	10	20	30	40	50	60	70	80	90	100
DCP	6	6	6	6	6	6	6	6	6	6	6
TAIC	2	2	2	2	2	2	2	2	2	2	2

## Data Availability

The authors declare that the main data supporting the findings and conclusions of this study are available within the article. Original and additional data is available from the corresponding author upon request.
